# Supplementary material for: Red Blood Cell Fatty Acid Patterns and Cognitive Functions in Adolescents: A Pooled Analyses with Two Cohort Study Data Sets
Source: Nutrients. 2025 Nov 5;17(21):3483. doi: 10.3390/nu17213483 (PMC12609364; doi:10.3390/nu17213483)
Supplement: Supplementary file 1 [file nutrients-17-03483-s001.zip › nutrients-3931818-supplementary - update.pdf]

Supplementary Table S1. Fatty acids of red blood cells and descriptive statistics.

**Fatty acid**	**Overall** [a] N= 660	**WSS** [a] N = 328	**INMA-Sabadell** [a] N = 332	**p-value** [b]
C14:0	1.09 (0.99)	1.54 (1.23)	0.64 (0.20)	<0.001
C16:0	19.75 (1.26)	20.09 (0.88)	19.41 (1.47)	<0.001
C16:1 n7	0.32 (0.16)	0.23 (0.15)	0.41 (0.11)	<0.001
C18:0	18.19 (1.22)	17.30 (0.90)	19.10 (0.72)	<0.001
All-trans C18:1	0.74 (0.47)	1.20 (0.12)	0.28 (0.10)	<0.001
C18:1 n-9 cis	15.11 (1.82)	16.18 (1.68)	14.03 (1.22)	<0.001
C18:2 n-6 cis	12.42 (1.51)	12.91 (1.48)	11.92 (1.36)	<0.001
C18:3 n-6	0.11 (0.08)	0.16 (0.08)	0.06 (0.02)	<0.001
C18:3 n-3	0.10 (0.06)	0.11 (0.08)	0.10 (0.03)	0.241
C20:0	0.21 (0.05)	0.24 (0.04)	0.18 (0.05)	<0.001
C20:1 n-9	0.31 (0.07)	0.27 (0.05)	0.35 (0.07)	<0.001
C20:2 n-6	0.34 (0.10)	0.30 (0.11)	0.37 (0.07)	<0.001
C20:3 n-6	1.72 (0.38)	1.81 (0.37)	1.64 (0.36)	<0.001
C20:4 n-6	17.55 (2.25)	15.76 (1.37)	19.35 (1.35)	<0.001
C20:5 n-3	0.35 (0.16)	0.31 (0.14)	0.40 (0.17)	<0.001
C22:0	0.29 (0.12)	0.39 (0.09)	0.18 (0.03)	<0.001
C22:4 n-6	3.40 (0.61)	3.24 (0.54)	3.57 (0.63)	<0.001
C22:5 n-3	1.40 (0.24)	1.35 (0.21)	1.45 (0.25)	<0.001
C22:5 n-6	0.61 (0.14)	0.63 (0.14)	0.59 (0.14)	<0.001
C22:6 n-3	3.99 (0.91)	3.96 (0.84)	4.02 (0.97)	0.433
C24:0	0.64 (0.25)	0.85 (0.19)	0.43 (0.05)	<0.001
C24:1 n-9	0.60 (0.29)	0.85 (0.19)	0.34 (0.06)	<0.001

WSS Walnuts Smart Snack Intervention Trial, INMA "Infancia y Medio Ambiente". Fatty acids are measured as the percentage of all fatty acids of red blood cells in each participant. This descriptive data corresponds to the complete rows (without missing values) used for matrix calculations in principal component analysis.

[a] Mean and standard deviation (SD).

[b] Wilcoxon rank sum test

Supplementary Table S2. Principal component, eigenvalues, and cumulative variance in principal components analysis.

PC	Eigenvalues	Variance (%)	Cumulative variance (%)
1	7.43	33.76	33.76
2	2.91	13.23	46.99
3	2.25	10.22	57.20
4	1.81	8.24	65.44
5	1.39	6.33	71.77
6	1.01	4.60	76.36
7	0.85	3.89	80.25
8	0.70	3.19	83.44
…			
22	0.01	0.03	100

PC Principal Component.

Supplementary Table S3. Multivariate regression models when exposure is in continuous tertiles (Tables 2 and 3) corrected p-values for multiple testing using the Benjamini-Hochberg false discovery rate.

Outcomes	Exposure	p-value [a]	rank	Benjamini-Hochberg critical (q-value)
PC3	Fluid Intelligence	0.003	1	0.004
PC3	CUPRAL	0.030	2	0.008
PC1	4 - back (d prime)	0.203	3	0.013
PC2	CUPRAL	0.116	4	0.017
PC3	CUPRAG	0.219	5	0.021
PC2	Fluid Intelligence	0.230	6	0.025
PC1	CUPRAG	0.229	7	0.029
PC2	4 - back (d prime)	0.180	8	0.033
PC1	Fluid Intelligence	0.500	9	0.038
PC1	CUPRAL	0.228	10	0.042
PC2	CUPRAG	0.354	11	0.046
PC3	4 - back (d prime)	0.575	12	0.050

PC1 very-long chain fatty acids, PC2 long-chain omega-6 fatty acids, PC3 omega-3 fatty acids, Fluid intelligence (PMA and Raven), CUPRAG roulettes task risk adjustment –Gain domain, CUPRAL roulettes task risk adjustment – loss domain, PC Principal Component

False Discovery Rate (FDR) significance threshold was defined at 0.05.

[a] p-value is p for trend.

Supplementary Table S4. Sensitivity analyses. Multiple linear regression models for fatty acids in principal component and cognitive function in the adolescent population.

Outcome/ Characteristic	Main model				Main model + variable			
	n	Beta [a]	95% CI [a]	p-value	n	Beta [b]	95% CI [b]	p-value
**PC1 "very-long chain fatty acids"**								
**Physical activity**								
4-back d prime	547	0.139	-0.060, 0.338	0.171	547	0.134	-0.065, 0.334	0.186
PMA and Raven	541	-0.077	-0.232, 0.078	0.328	541	-0.078	-0.233, 0.077	0.325
CUPRAL	551	-0.188	-0.582, 0.206	0.349	551	-0.189	-0.583, 0.206	0.348
CUPRAG	551	-0.199	-0.607, 0.208	0.337	551	-0.197	-0.606, 0.211	0.343
**MedDiet**								
4-back d prime	541	0.144	-0.057, 0.345	0.159	541	0.158	-0.042, 0.359	0.122
PMA&Raven	535	-0.089	-0.244, 0.066	0.258	535	-0.075	-0.230, 0.079	0.339
CUPRAL	546	-0.204	-0.600, 0.193	0.313	546	-0.198	-0.596, 0.200	0.329
CUPRAG	546	-0.201	-0.608, 0.206	0.333	546	-0.204	-0.613, 0.205	0.327
**Mother mental disorder**								
4-back d prime	530	0.138	-0.063, 0.338	0.178	530	0.137	-0.063, 0.338	0.178
PMA and Raven	524	-0.042	-0.199, 0.116	0.602	524	-0.042	-0.200, 0.115	0.599
CUPRAL	534	-0.175	-0.579, 0.228	0.394	534	-0.171	-0.573, 0.231	0.403
CUPRAG	534	-0.167	-0.587, 0.252	0.434	534	-0.165	-0.585, 0.254	0.439
**Maternal social class**								
4-back d prime	523	0.125	-0.075, 0.325	0.219	523	0.125	-0.074, 0.324	0.219
PMA and Raven	516	-0.082	-0.240, 0.075	0.306	516	-0.084	-0.241, 0.072	0.292
CUPRAL	527	-0.179	-0.578, 0.219	0.378	527	-0.183	-0.581, 0.214	0.366
CUPRAG	527	-0.232	-0.647, 0.183	0.273	527	-0.240	-0.649, 0.170	0.251
**PC2 "long chain omega-6 fatty acids"**								
**Physical activity**								
4-back d prime	547	0.079	-0.054, 0.212	0.242	547	0.078	-0.055, 0.211	0.249
PMA and Raven	541	-0.068	-0.174, 0.038	0.208	541	-0.066	-0.172, 0.040	0.225
CUPRAL	551	-0.079	-0.345, 0.187	0.560	551	-0.074	-0.340, 0.192	0.586
CUPRAG	551	-0.182	-0.457, 0.093	0.193	551	-0.182	-0.458, 0.093	0.194
**MedDiet**								
4-back d prime	541	0.080	-0.053, 0.214	0.239	541	0.096	-0.038, 0.230	0.160
PMA and Raven	535	-0.074	-0.179, 0.032	0.170	535	-0.060	-0.165, 0.046	0.268
CUPRAL	546	-0.089	-0.356, 0.178	0.515	546	-0.083	-0.352, 0.186	0.547
CUPRAG	546	-0.178	-0.452, 0.096	0.202	546	-0.183	-0.459, 0.093	0.193
**Mother mental disorder**								
4-back d prime	530	0.104	-0.031, 0.238	0.130	530	0.104	-0.031, 0.239	0.130
PMA and Raven	524	-0.062	-0.170, 0.047	0.265	524	-0.060	-0.169, 0.048	0.276
CUPRAL	534	-0.094	-0.369, 0.181	0.504	534	-0.103	-0.377, 0.171	0.462
CUPRAG	534	-0.187	-0.472, 0.099	0.199	534	-0.191	-0.477, 0.095	0.190
**Maternal social class**								
4-back d prime	523	0.081	-0.053, 0.215	0.233	523	0.082	-0.052, 0.216	0.229
PMA and Raven	516	-0.072	-0.180, 0.037	0.194	516	-0.077	-0.184, 0.031	0.162
CUPRAL	527	-0.145	-0.415, 0.126	0.295	527	-0.156	-0.426, 0.114	0.258
CUPRAG	527	-0.193	-0.475, 0.089	0.179	527	-0.218	-0.496, 0.060	0.124

PC principal component, CI confidence interval, MedDiet Mediterranean Diet, CUPRAG roulettes task risk adjustment – gain domain; CUPRAL roulettes task risk adjustment – loss domain.

[a] Beta coefficient (slope) and 95% CI (Confidence Interval) estimated using multiple linear regression models adjusted for sex, age, maternal education, BMI z-score and cohort. The exposure variable is principal component in continuous tertiles (1, 2, and 3).

[b] Beta coefficient (slope) and 95% CI (Confidence Interval) estimated using multiple linear regression models adjusted for sex, age, maternal education, BMI z-score and cohort. The exposure variable is principal component in continuous tertiles

(1, 2, and 3). The models were additionally adjusted physical activity or adherence to the Mediterranean Diet or mother mental disorder or maternal social class.

Supplementary Table S5. Sensitivity analyses. Multiple linear regression models for fatty acids in principal component and cognitive function in the adolescent population.

Outcome/ Characteristic	Main model				Main model + variable			
	n	Beta [a]	95% CI [a]	p-value	n	Beta [b]	95% CI [b]	p-value
PC3 "Omega-3 fatty acids"								
**Physical activity**								
4-back d prime	547	0.040	-0.083, 0.163	0.523	547	0.036	-0.087, 0.159	0.567
PMA and Raven	541	0.137	0.041, 0.233	0.005	541	0.136	0.040, 0.231	0.006
CUPRAL	551	0.150	-0.094, 0.395	0.228	551	0.146	-0.099, 0.391	0.243
CUPRAG	551	0.276	0.024, 0.528	0.032	551	0.279	0.026, 0.531	0.031
**MedDiet**								
4-back d prime	541	0.049	-0.075, 0.173	0.437	541	0.033	-0.092, 0.158	0.603
PMA and Raven	535	0.131	0.035, 0.227	0.007	535	0.118	0.022, 0.214	0.016
CUPRAL	546	0.137	-0.109, 0.384	0.275	546	0.132	-0.117, 0.381	0.299
CUPRAG	546	0.243	-0.009, 0.495	0.059	546	0.250	-0.005, 0.505	0.054
**Mother mental disorder**								
4-back d prime	530	0.027	-0.097, 0.151	0.669	530	0.027	-0.097, 0.151	0.670
PMA and Raven	524	0.125	0.027, 0.223	0.012	524	0.125	0.027, 0.222	0.012
CUPRAL	534	0.157	-0.094, 0.408	0.220	534	0.159	-0.091, 0.410	0.212
CUPRAG	534	0.250	-0.011, 0.510	0.060	534	0.251	-0.010, 0.512	0.059
**Maternal social class**								
4-back d prime	523	0.055	-0.068, 0.179	0.378	523	0.050	-0.073, 0.173	0.427
PMA and Raven	516	0.133	0.036, 0.231	0.007	516	0.123	0.025, 0.220	0.014
CUPRAL	527	0.173	-0.074, 0.421	0.170	527	0.168	-0.079, 0.415	0.182
CUPRAG	527	0.249	-0.009, 0.506	0.058	527	0.222	-0.032, 0.477	0.087

PC principal component, CI confidence interval, MedDiet Mediterranean Diet, CUPRAG roulettes task risk adjustment – gain domain; CUPRAL roulettes task risk adjustment – loss domain.

[a] Beta coefficient (slope) and 95% CI (Confidence Interval) estimated using multiple linear regression models adjusted for sex, age, maternal education, BMI z-score and cohort. The exposure variable is principal component in continuous tertiles (1, 2, and 3).

[b] Beta coefficient (slope) and 95% CI (Confidence Interval) estimated using multiple linear regression models adjusted for sex, age, maternal education, BMI z-score and cohort. The exposure variable is principal component in continuous tertiles (1, 2, and 3). The models were additionally adjusted physical activity or adherence to the Mediterranean Diet or mother mental disorder or maternal social class.

Supplementary Table S6. Interaction. Multiple linear regression models for fatty acids in principal component and cognitive function in the adolescent population.

Characteristic	n	Beta [a]	95% CI [a]	p-value
**"PC1 very-long chain fatty acids"**				
4 d prime				
PC1	568	0.04	-0.24, 0.31	0.788
PC1 * walnuts	311	0.17	-0.21, 0.55	0.388
PMA and Raven				
PC1	562	-0.09	-0.32, 0.13	0.410
PC1 * walnuts	315	0.08	-0.22, 0.38	0.619
CUPRAG				
PC1	572	-0.36	-0.91, 0.20	0.209
PC1 * walnuts	315	0.19	-0.57, 0.95	0.621
CUPRAG				
PC1	572	-0.08	-0.65, 0.50	0.794
PC1 * walnuts	315	-0.32	-1.1, 0.47	0.433
**PC2 "long-chain omega-6 fatty acids"**				
4 d prime				
PC2	568	0.04	-0.15, 0.24	0.657
PC2 * WSS	311	0.08	-0.18, 0.34	0.548
PMA and Raven				
PC2	562	-0.13	-0.29, 0.03	0.107
PC2 * WSS	315	0.12	-0.09, 0.32	0.273
CUPRAG				
PC2	572	-0.27	-0.67, 0.12	0.171
PC2 * WSS	315	0.29	-0.23, 0.81	0.271
CUPRAL				
PC2	572	-0.37	-0.78, 0.04	0.074
PC2 * WSS	315	0.27	-0.26, 0.81	0.319
**PC3 "omega-3 fatty acids"**				
4 d prime				
PC3	568	-0.03	-0.21, 0.14	0.717
PC3 * WSS	311	0.12	-0.12, 0.36	0.316
PMA and Raven				
PC3	562	0.18	0.04, 0.32	0.014
PC3 * WSS	315	-0.06	-0.25, 0.12	0.506
CUPRAG				
PC3	572	0.06	-0.30, 0.42	0.742
PC3 * WSS	315	0.12	-0.35, 0.60	0.613
CUPRAL				
PC3	572	0.22	-0.15, 0.59	0.246
PC3 * WSS	315	0.10	-0.39, 0.59	0.694

PC principal component, CI confidence interval, MedDiet Mediterranean Diet, CUPRAG roulettes task risk adjustment – gain domain; CUPRAL roulettes task risk adjustment – loss domain.

[a] Beta coefficient (slope) and 95% CI (Confidence Interval) estimated using multiple linear regression models adjusted for sex, age, maternal education, BMI z-score, cohort and (cohort x PC). The exposure variable is principal component in continuous tertiles (1, 2, and 3).

Supplementary Figure S1. Correlation of fatty acids in WSS and INMA-Sabadell cohort population.

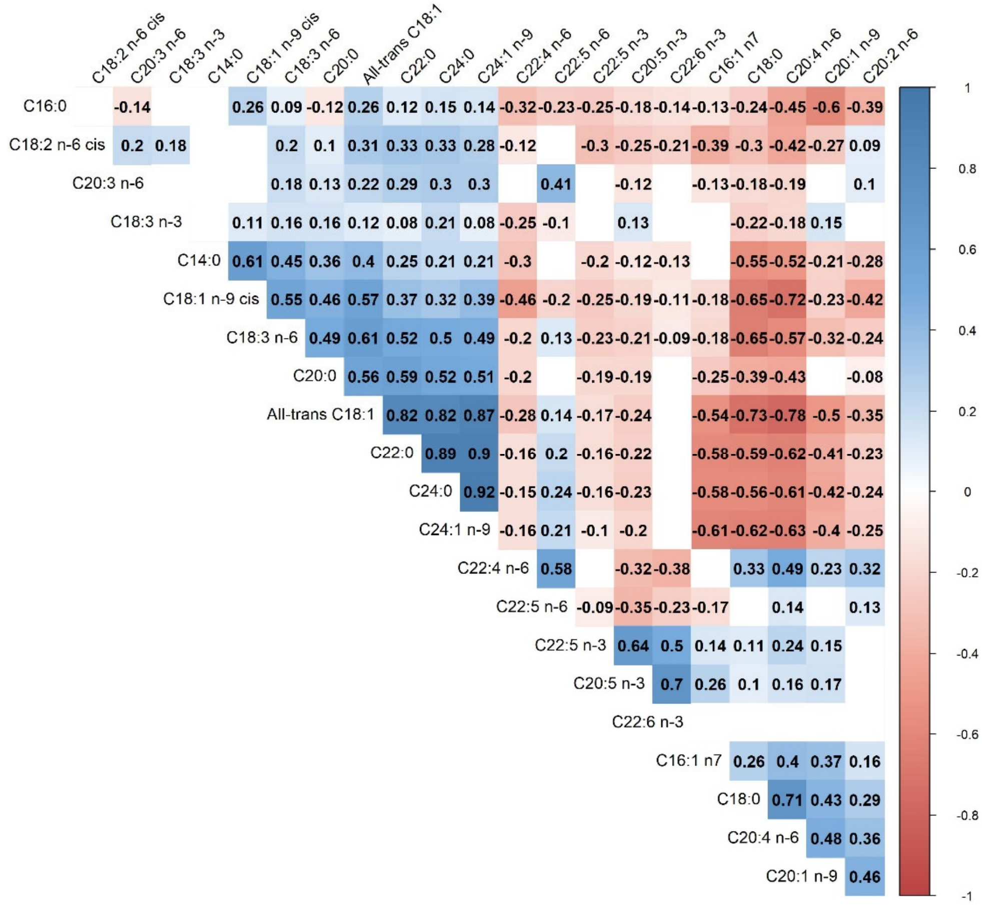

Spearman correlation is reported with a significance level of $p < 0.05$. Only statistically significant association are shown in the correlation map.

Supplementary Figure S2. Scree plot for principal component analysis on fatty acids matrix.

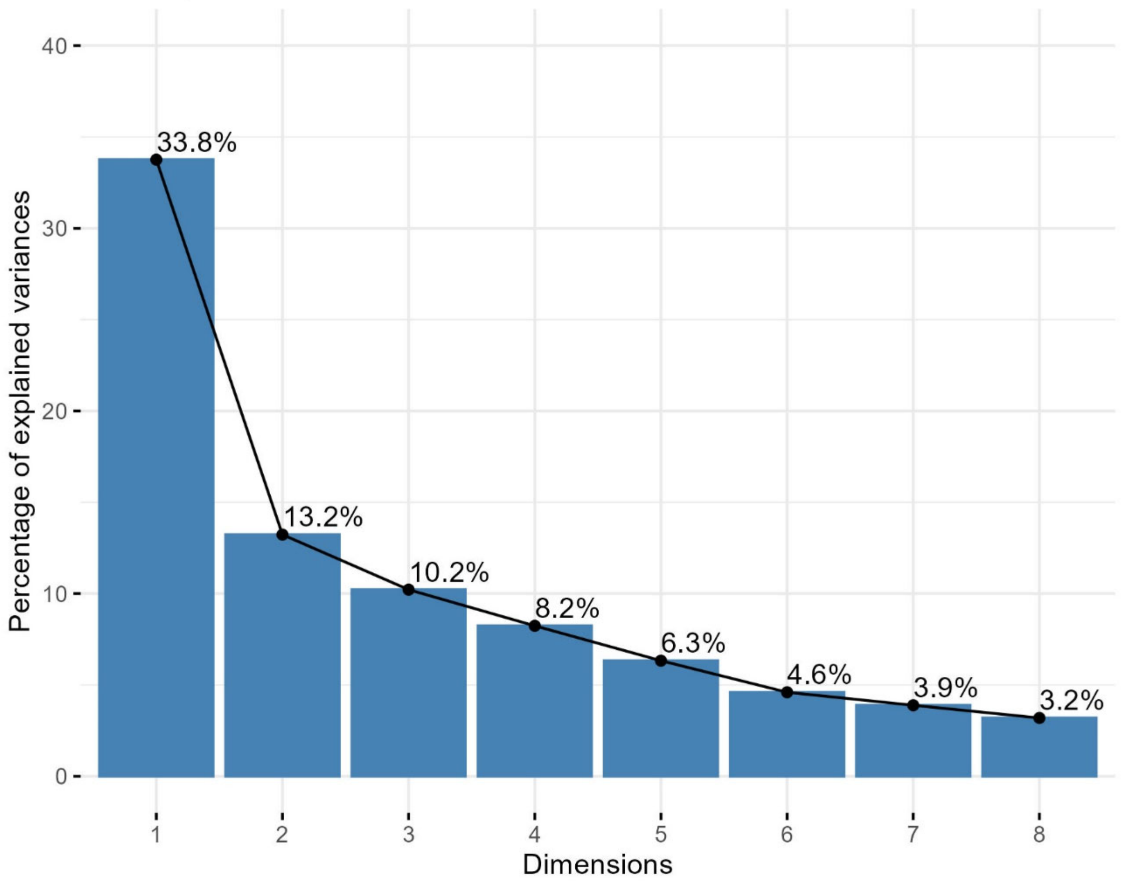

Supplementary Figure S3. Biplot for principal component 1, 2 and 3.

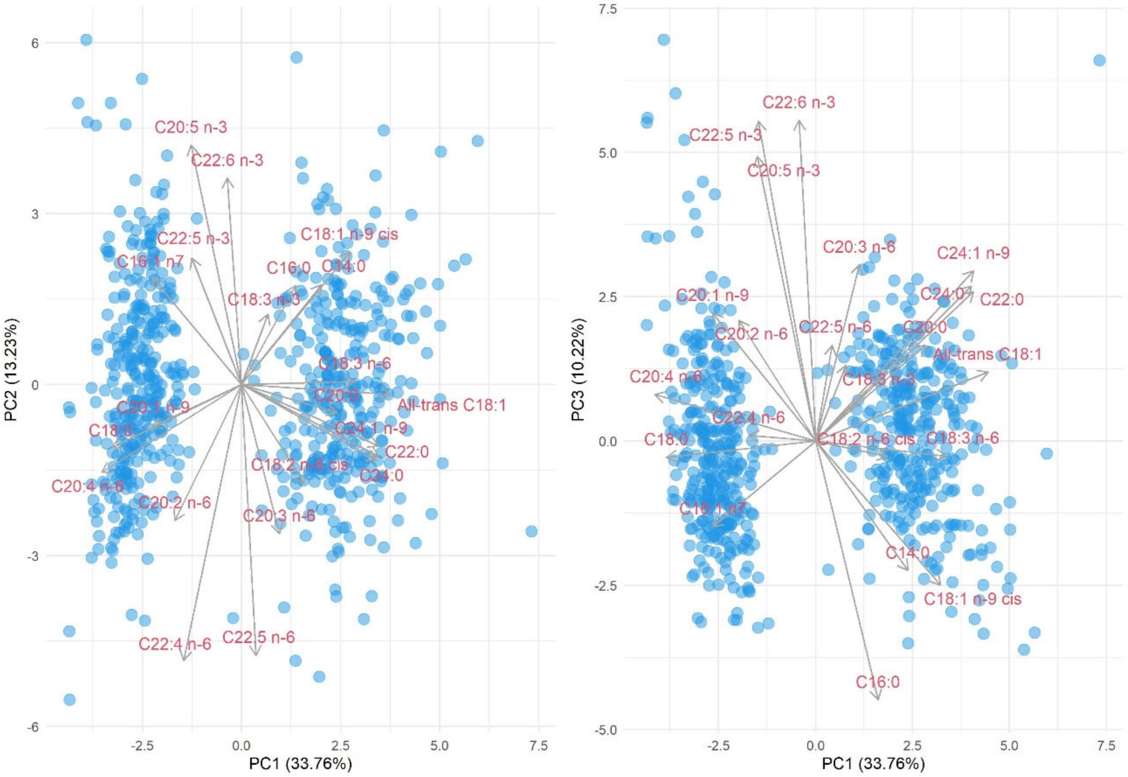

PC Principal Component.

PC1 very-long chain fatty acids, PC2 long-chain omega-6 fatty acids, PC3 omega-3 fatty acids.

Supplementary Figure S4. Batch effect and Procrustes analyses of Principal Component Analyses.

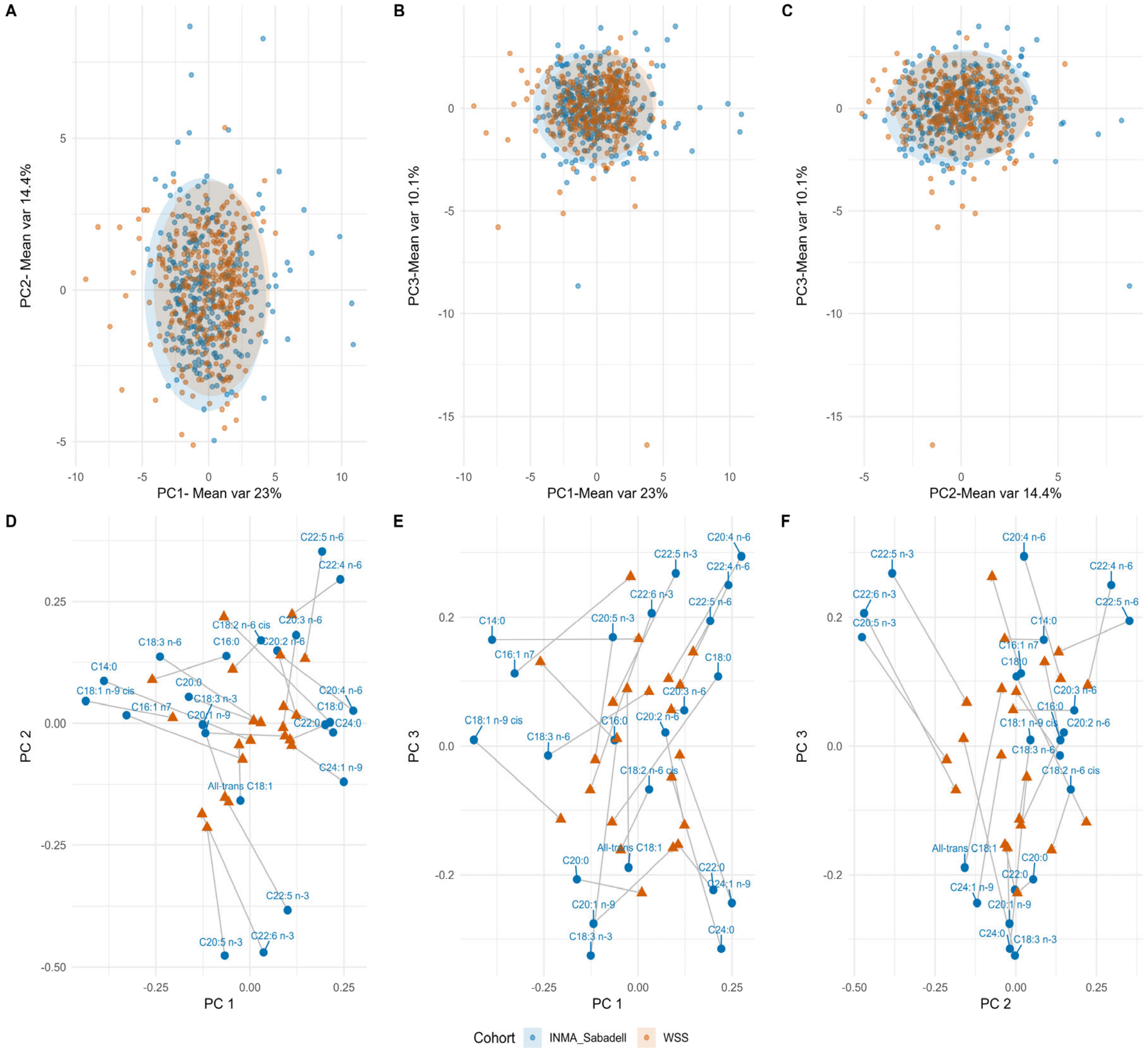

PC1 very-long chain fatty acids, PC2 long-chain omega-6 fatty acids, PC3 omega-3 fatty acids.

Panels A, B, and C display the unrotated Principal Component (PC) scores used to visualize the batch effect (difference between cohorts) in three dimensions: PC1 vs. PC2 (A), PC1 vs. PC3 (B), and PC2 vs. PC3 (C). The cohorts are separated by color (WSS and INMA-Sabadell). Panels D, E, and F show the optimal superimposition of the PCA loadings using Procrustes analysis for the same three PC combinations from WSS and INMA-Sabadell datasets. The reference loadings (WSS, circles) are connected by gray lines to the optimally rotated and scaled target loadings (INMA-Sabadell, triangles). The Procrustes Root Mean Squared Error (RMSE) for the combined 3D structure is 0.294 (IQR: 0.21, 0.34). The optimal transformation required a Scaling Factor of 0.59 for the target cohort (INMA-Sabadell) when WSS was used as a reference dataset. The largest residual distances (longest gray vectors) in the Procrustes analysis projected onto the PC1 vs.PC2

plane were concentrated in C14:0 (distance: 0.403), followed by the long-chain omega-3 PUFAs (C22:6 n-3: 0.320 and C20:5 n-3: 0.267)
